# Foreign Body Moves Retrograde through Ileocecal Valve during Colonoscopy

**DOI:** 10.1155/2017/8707959

**Published:** 2017-05-17

**Authors:** Maria Paparoupa, Markus Bruns-Toepler

**Affiliations:** Department of Pulmonary Diseases, Infectious Diseases, Gastroenterology, Nephrology and Intensive Care Unit, University Hospital of Giessen, Klinikstr. 33, 35392 Giessen, Germany

## Abstract

Ingestion of foreign bodies and particularly of button or/and cylindrical batteries is frequent in children and adults with underlying psychiatric diseases. We present a case of a 30-year-old woman with unstable borderline disorder, where overall 4 button and 2 cylindrical batteries were endoscopically removed from her digestive system. During the last session of colonoscopy a peculiar incident was observed, as a cylindrical battery of 15 mm diameter and 43 mm length moved retrograde through ileocecal valve into the small bowel. The foreign body removal from terminal ileum was effective and safe using an endoscopic loop. This report suggests that endoscopic insertion in terminal ileum should be attempted in every colonoscopy session conducted under the indication of foreign body removal, as the possibility of retrograde movement of even large foreign bodies in the colon and through ileocecal valve is given.

## 1. Introduction

Ingestion of foreign bodies and particularly of button or/and cylindrical batteries is frequent in children and scientific literature is mainly based on pediatric case series [1]. Nevertheless, the phenomenon is also observed in adults, more often in psychiatric patients suffering of unstable borderline disorder. Several reports describe the ingestion of multiple batteries as suicidal attempt or an act aiming to relieve sudden emotional discomfort on this population [2]. The clinical features can be harmless until foreign bodies are disposed through the natural way or take the form of major complications such as esophageal perforation [3], aortic-esophageal fistula [3], tracheoesophageal fistula [4], bowel obstruction or perforation [2] and even spondylodiscitis [5], and airway impaction [6]. Endoscopic treatment is preferred as high rates of removal success, low incidence of complications, and reduced hospitalization make it favorable towards surgical approach [7]. We present a case of endoscopic removal of overall 4 button and 2 cylindrical batteries from the digestive system of a young woman, where a peculiar incident of a foreign body moving retrograde through ileocecal valve was observed.

## 2. Case Presentation

The 30-year-old woman was admitted to our intensive care unit after being endotracheal intubated in emergency setting due to possible battery ingestion. The patient was suffering from a severe borderline personality disorder with a long medical history of recurrent suicide attempts and numerous incidents of nonsuicidal self-injury (NSSI) causing deep scarves on her arms and breasts. She was living in a residence specialized on giving care that ensures the surveillance of members with refractory psychiatric disorders who were unable to get integrated into community. Approximately one hour before her admission the patient called social servers for help and confessed having swallowed an uncertain number of different types of batteries while being unattended. Although relief of sudden emotional stress with no suicidal attention was her given motive, the patient refused to follow emergency physician to the hospital and became violent, which led to application of sedative-hypnotic drugs and subsequently to prehospital endotracheal intubation.

**Figure 1:**
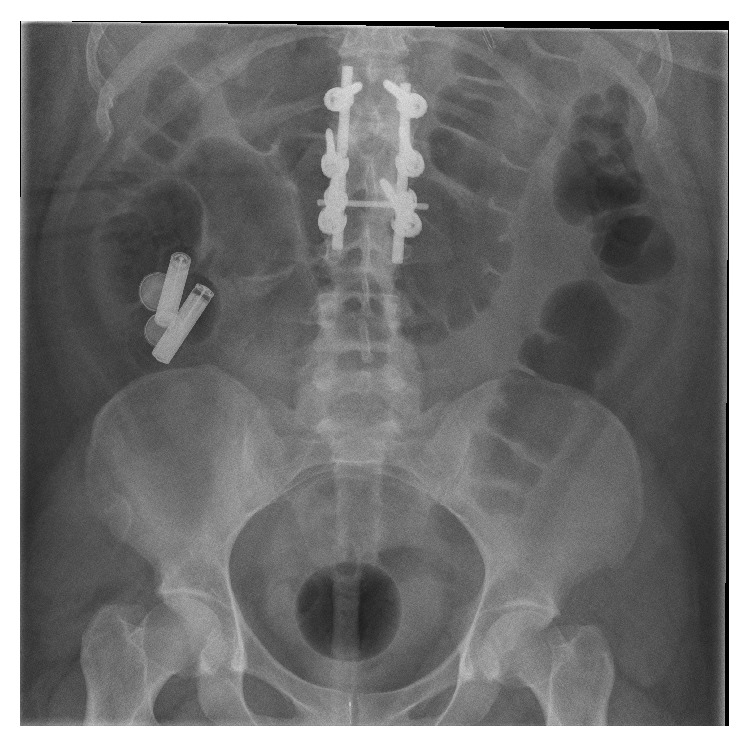
X-ray presenting a cluster of four batteries, two button batteries, and two cylindrical ones in Right Lower Quadrant (RLQ) of abdomen.

The general physical examination on the board revealed signs of acute abdomen and laboratory tests were unremarkable. An immediate upper intestinoscopy was undertaken in order to exclude esophageal impaction and two button batteries were removed from the stomach of the patient. Preparation for colonoscopy was initiated. As the total number of digested foreign bodies remained uncertain an X-ray of abdomen was performed 12 hours after starting colon prep. As shown in Figure 1 two more button batteries and two cylindrical ones were found to form a cluster in Right Lower Quadrant (RLQ) of abdomen. Having concerns that the batteries were already oxidated or even stuck in terminal ileum, we proceeded immediately with lower intestinal endoscopy.

**Figure 2:**
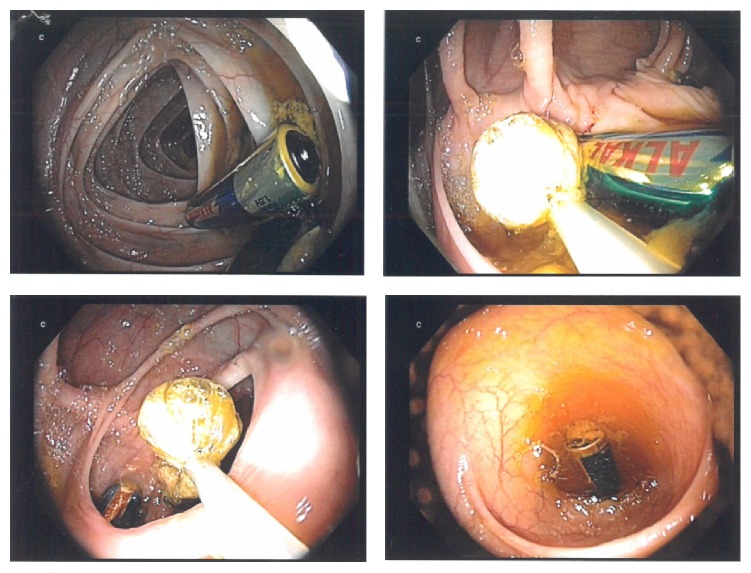
Having captured the third battery in a Dormia basket, we could see the last cylindrical one, in the background of endoscopic field deep in cecum.

**Figure 3:**
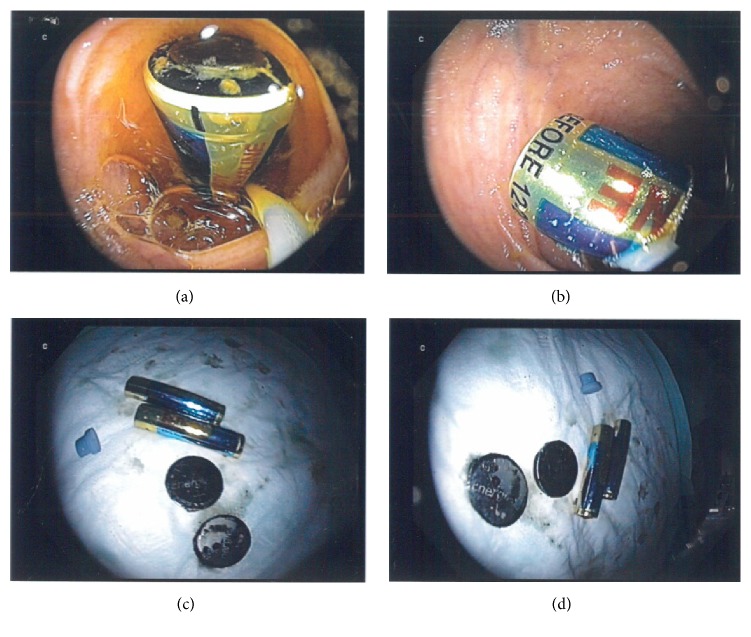
The cylindrical battery was detected in terminal ileum, grasped with an endoscopic loop and pulled through the valve ((a) and (b)). All four batteries are presented after their removal per anus ((c) and (d)).

**Figure 4:**
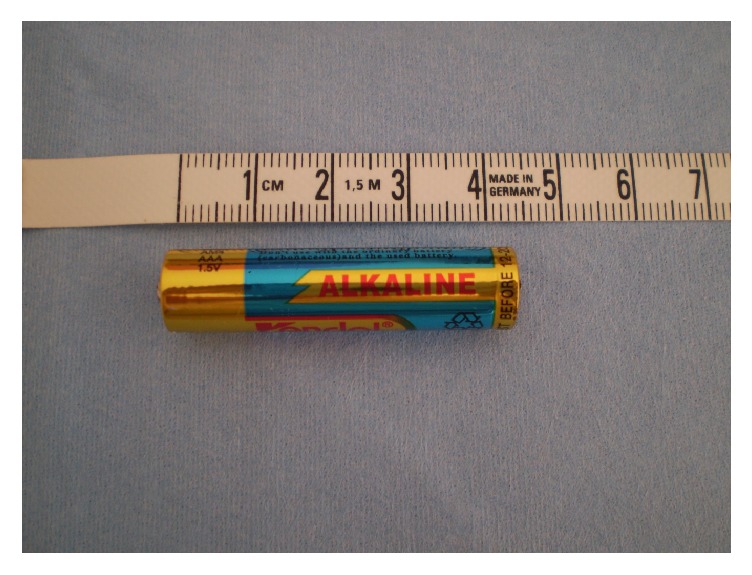
The length of the cylindrical battery was more than 4 cm.

Under the implementation of endoscopic loops and Dormia basket, three of overall four batteries were removed from cecum as they have already passed into colon. Having captured the third one, we could see the last cylindrical battery in the background of endoscopic field deep in cecum (Figure 2). Surprisingly after inserting colon again in order to remove it, the foreign body was not detectable anymore. The examination was unsuccessfully repeated over three sessions with careful inspection of the colonic loops from cecum to rectum. We supposed subsequently that the battery could have moved retrograde into small bowel. We negotiated colonoscope through the ileocecal valve, detected the foreign body in terminal ileum, grasped it with an endoscopic loop, pulled it through the valve, and removed it per anus (Figure 3). The length of the battery was more than 4 cm (Figure 4) with a diameter of 15 mm.

## 3. Discussion

There have been reports of small bowel obstruction secondary to battery ingestion, which have been managed surgically [2]. In our case two button and two cylindrical batteries passed through ileocecal valve, although radiological assessment of their exact position in the bowel was difficult and we had to proceed with endoscopic evaluation. Our main concern was the oxidation of the button batteries as several hours ago two of such foreign bodies were removed from the stomach of the patient and we could already macroscopically detect multiple oxidative erosions on their surface. Unfortunately no picture documentation of the upper intestinoscopy of this case has been preserved. Release of toxic metals in the gastrointestinal system can lead to intoxication through heavy metals like Cadmium (Cd), Hydrargyrum (Hg), and others, as elsewhere described in the literature [8]. Another factor which accelerated our decision to perform endoscopic removal of the batteries, though missing signs of small bowel obstruction, was the reported possibility of bowel perforation, as batteries interact with each other and electrical discharge, leading to severe tissue damage, can be produced. According to a retrospective review of case series, the anatomic direction of the battery's negative pole was a predictive risk factor of long term complications due to button battery impaction [9]. The experimental work of Yasui in rats showed that, prior to leakage of the cell alkali, ulceration or perforation of the digestive mucosa occurred due to electric discharge of the battery and this effect was independent of fasted state or acidic environment [10].

In our case, after uncomplicated colonoscopic removal of two button and one cylindrical batteries, the retrograde movement of the last cylindrical one through ileocecal valve was observed. We suppose that a foreign body of such diameter (43 mm) and length (15 mm) was able to move backwards through a tight pathway being forced by intestinal peristaltic waves and elevated liquid and air pressure in colon during colonoscopy session. The retrograde movement of a battery in gastrointestinal system has been described before in a case where battery has moved from the stomach to esophagus after administration of ipecac [1]. To our knowledge, this is the first report of a retrograde movement through ileocecal valve. Colonoscopic removal of the foreign body from terminal ileum was effective and safe. Tissue damage, perforation, and small bowel obstruction could be prevented. The postinterventional course of the patient was uneventful and normal bowel activity was present within few hours. This report suggests that endoscopic insertion in terminal ileum should be attempted in every colonoscopy session conducted under the indication of foreign body removal, as the possibility of retrograde movement of even large foreign bodies in the colon and through ileocecal valve is given.
